# Tunable CAR-NK-92 activity in the tumor microenvironment via a dual ATF4-responsive circuit

**DOI:** 10.3389/fimmu.2026.1792164

**Published:** 2026-03-05

**Authors:** Enzo Manchon, Nell Hirt, Aravindhan Soundiramourty, Benjamin Versier, Yves Christen, Dominique Charron, Jacques Mallet, Nabila Jabrane-Ferrat, Che Serguera, Reem Al-Daccak

**Affiliations:** 1 National Institute of Health and Medical Research (INSERM) UMR 1342, Paris University, Saint-Louis Hospital, Paris, France; 2 Asfalia Biologics, Paris Brain Institute (ICM), Hôpital Pitié-Salpêtrière, Paris, France; 3 Institute for Infectious and Inflammatory Diseases, National Center of Scientific Reserach (CNRS) UMR5051, Institute of Health and Medical Research (INSERM) UMR1291, Toulouse University, Toulouse, France

**Keywords:** CAR-NK cell therapy, ER stress-inducing drugs, FDA-approved NK-92 cells, GCN2-ATF4 pathway, regulatable systems, solid tumors, tumor microenvironment

## Abstract

Chimeric antigen receptor–engineered NK-92 (CAR-NK-92) cells emulate activated natural killer cells, combining potent innate cytotoxicity with CAR-driven antigen specificity. Their scalability and FDA approval make them attractive for universal use. However, their application in solid tumors remains limited by the immunosuppressive tumor microenvironment (TME), which is often characterized by hypoxia and nutrient deprivation. We recently demonstrated that an ATF4-inducible promoter, 2xAARE-YB, enables spatial and temporal control of CAR expression in T cells, enhancing safety by restricting expression to amino acid-deprived TME while reducing exhaustion to improve persistence. In this study, we adapted the 2xAARE-YB system for CAR-NK-92 cells. Under glucose-limited conditions, a hallmark of the TME, the system effectively regulated CAR expression, enabling potent antigen-specific cytotoxicity. In xenograft models, the nutrient-responsive 2xAARE-YB system achieved regulated intratumoral CAR expression *in vivo*, supporting its potential for the development of safer therapeutic strategies. Additionally, the clinically approved ER stress–inducing drug artesunate also reliably activated the circuit, offering a drug-inducible regulation of CAR expression. Collectively, these findings establish 2xAARE-YB as a dual-mode regulatory platform that enables tunable, context-dependent CAR expression in NK-92 cells. Although this approach may be more effective in HLA I-negative tumors than in HLA I-positive tumors, it represents a promising path toward safer and more adaptable CAR-NK-92 therapies tailored for the dynamic metabolic constraints of solid tumors.

## Introduction

Although chimeric antigen receptor (CAR) T cell therapy has achieved remarkable clinical success, CAR-engineered natural killer (CAR-NK) cells are emerging as a promising alternative with several distinct advantages (1). NK cells possess inherent antitumor activity through receptor-ligand recognition and antibody-dependent cellular cytotoxicity. Unlike T cells, NK cells lack polyclonal T cell receptor, thereby reducing the risk of graft-versus-host disease in allogeneic settings. Moreover, they exhibit a cytokine profile associated with lower toxicity, translating to a reduced risk of cytokine release syndrome (CRS) (2). The NK-92 cell line, which closely replicates activated NK cells with robust cytotoxic function, supports large-scale expansion suitable for clinical-grade applications and has received FDA approval for clinical use. Thus, it enables a uniform and highly cytotoxic cost-effective off-the-shelf CAR-NK therapies (3).

Despite this promise, CAR-NK-92 cell therapies face significant challenges in the context of solid tumors, including limited persistence and suppression by the tumor microenvironment (TME), which is typically characterized by hypoxia and nutrient deprivation (1). Although CAR-NK cells may present a lower risk of on-target off-tumor toxicity compared to CAR-T cells, safety remains a critical concern (4). Current strategies to confine CAR activity to tumor sites or deactivate CARs upon off-target engagement often rely on single-mode regulatory systems, which may be inadequate in the heterogeneous and dynamic TME (5, 6). To address these limitations and improve spatiotemporal control of CAR expression, dual-regulatory systems responsive to TME stress are under investigation (7, 8).

One promising approach explores Activating Transcription Factor-4 (ATF4) pathway, which is activated under nutrient deprivation and endoplasmic reticulum (ER) stress via PKR-like endoplasmic reticulum kinase (PERK). We recently demonstrated that an ATF4-inducible promoter, 2xAARE-YB, can restrict CAR expression in T cells under amino acid-limiting conditions, thereby enhancing safety and persistence (8). In this study, we deploy the innovative 2xAARE-YB system to drive selective CAR-NK-92 activation in the solid tumor microenvironment. Under glucose-limited conditions, another hallmark of the TME, the system effectively regulated CAR expression in NK-92 cells, while preserving potent antitumor cytotoxicity. Furthermore, the clinically approved ER stress-inducing drug Artesunate activated the same pathway, thereby enabling drug-inducible CAR expression in these cells.

Collectively, our findings establish 2xAARE-YB as a dual-mode, TME-responsive platform enabling precise control of CAR expression in NK-92 cells, offering a promising strategy for the development of safer, and more adaptable CAR-NK-92 therapies in solid tumors.

## Materials and methods

Detailed description of reagents and protocols are provided under **Supplementary Material** and Tables.

### Cell lines and mice

NK-92 (ACC-488, DSMZ), engineered to constitutively express IL-15, were maintained in RPMI-1640 medium supplemented with 20% Fetal Bovine Serum (FBS), GlutaMAX and penicillin-streptomycin (Gibco, Fisher Scientific, France). MDA-MB 231 breast cancer cells (HTB-26, ATCC) and ESTDAB-109 melanoma cells (EST-109 (UKRV-Mel-2); a gift from Federico Garrido, Granada University) were maintained in RPMI-1640 with 10% FBS, GlutaMAX and penicillin-streptomycin. All cell lines were regularly tested for Mycoplasma using the MycoBlue Mycoplasma Detector (Vazyme, Biovalley, France). Male NXG (NOD-*Prkdc*^scid^ -*IL2rg*^Tm1^) mice (Janvier Labs, France) aged 6–8 weeks and weighing 20-22g were kept under pathogen-free conditions.

### CAR constructs and lentiviral production and transduction

Self-inactivated (SIN) lentiviral vectors were constructed by inserting synthesized or PCR-amplified [2xAARE-YB-transgene (± polyA)] cassettes into destination plasmids using NEBuilder HiFi DNA Assembly (New England Biolabs, France). Anti-CD19-41BBζ-CAR cDNA (8) was driven by either EF1α or 2xAARE-YB promoters. Lentiviral particles were produced in HEK-293T cells (CRL-11268, ATCC) using a standard second-generation packaging system, as previously described (9), and titrated via p24 ELISA (Zeptometrix, #0801008), assuming 1 pg p24 = 100 TU. NK-92 cells were transduced with a promoter-less TRAP-IL-15 vector, enabling constitutive IL-15 expression controlled endogenously by a cellular promoter, and with 2xAARE-YB-CAR or EF1α-CAR lentiviral vectors encoding anti-CD19-41BBζ-CAR. EST-109 and MDA-MB 231 cells were transduced with a TRAP-CD19 vector carrying a splice acceptor–T2A–CD19–P2A–bsd cassette, enabling constitutive CD19-expression.

### *In vitro* experimental protocol

Transduced cells or control cells were washed and resuspended at 2x10^5^ cells/mL in either RPMI-1640 medium selectively depleted of arginine, leucine, lysine, methionine, glutamine, tryptophan, or glucose, and supplemented with 5% FBS, GlutaMAX (except for glutamine-restricted condition), penicillin-streptomycin, IL-2 (Miltenyi Biotec, France) and 1/1000 (amino acids) or 1/100 (glucose), or in control RPMI-1640 supplemented with 10% FBS, L-glutamine, penicillin-streptomycin and IL-2. For hypoxic conditions, cells were incubated in complete medium within hypoxia chamber (Stemcell technologies, France). Cells were then harvested for further experiments including RT-qPCR, western blot, and flow cytometry.

### 2D-cytotoxicity assay

CD19^--^and CD19^+^-cancer cells (5x10^3^ each) were co-cultured with CAR-NK-92 cells (2x10^4^) in control, glucose-restricted medium, or in artesunate-supplemented control media. Artesunate was used at 1 µM, a concentration selected based on established human pharmacokinetic studies demonstrating that peak plasma levels following intravenous administration reach the low micromolar range (10), placing 1 µM within clinically achievable exposure levels. Target cells were detached using 0.05% Trypsin-EDTA (Gibco), acquired on a BD Canto II (BD biosciences) using FACS Diva software, and analyzed with FlowJo (10.8.1) software. Specific lysis (%) was calculated as: (1-(control ratio/experimental ratio) x 100), where the control ratio corresponds to target cells co-cultured with untransduced (mock) NK-92 cells.

### 3D-spheroid assay

Spheroids of EST-109 melanoma and MDA-MB 231 breast cancer cells constitutively expressing GFP, were formed over 4 days in RPMI-1640 supplemented with 5% FBS, GlutaMAX, penicillin-streptomycin and 2.5% Matrigel Basement Membrane Matrix (Corning). Spheroid were then transferred to round-bottom 96-well plates containing control medium, glucose-restricted medium or control medium with artesunate (1 μM). NK-92 cells (mock, 2xAARE-YB-CAR, or EF1α-CAR; 20x10³ cells per well) were subsequently added to each spheroid. Spheroid fluorescence was imaged every 2 hours using a 10X objective on the Incucyte live-cell imaging system (Essen Bioscience) under standard culture conditions (37 °C, 5% CO_2_). The size of the largest GFP object was quantified using Incucyte software (Essen Bioscience). All data were normalized to the initial time point and expressed as fold change of the largest GFP object over time.

### *In vivo* protocols

Mice were subcutaneously (s.c.) injected with 2x10^6^ CD19^+^-EST-109 cells in PBS. The indicated doses of mock, 2xAARE-YB-CAR or EF1a-CAR NK-92 cells were intratumorally (i.t.) injected in PBS. At experimental endpoint, mice were sacrificed, then tumors were excised and kept in RPMI-1640 with 10% FBS. Tumors were mechanically dissociated and digested in Collagenase IV and DNase I. Single-cell suspensions were then prepared by filtering through a 70μm cell strainer and processed for flow cytometry analysis.

### Statistics

Statistical analyses were conducted using GraphPad Prism 10. Sample size and details of plot descriptions are provided respective figure legends. Statistical variances between two groups were assessed using either an unpaired student’s t-test or a Mann-Whitney U test as appropriate. For comparisons involving three or more groups, analysis of variance (one-way or two-way ANOVA) was used followed by appropriate multiple comparison tests. Statistical significance was denoted as p < 0.05.

## Results

### ATF4 pathway activation in NK-92 cells

**Figure 1:**
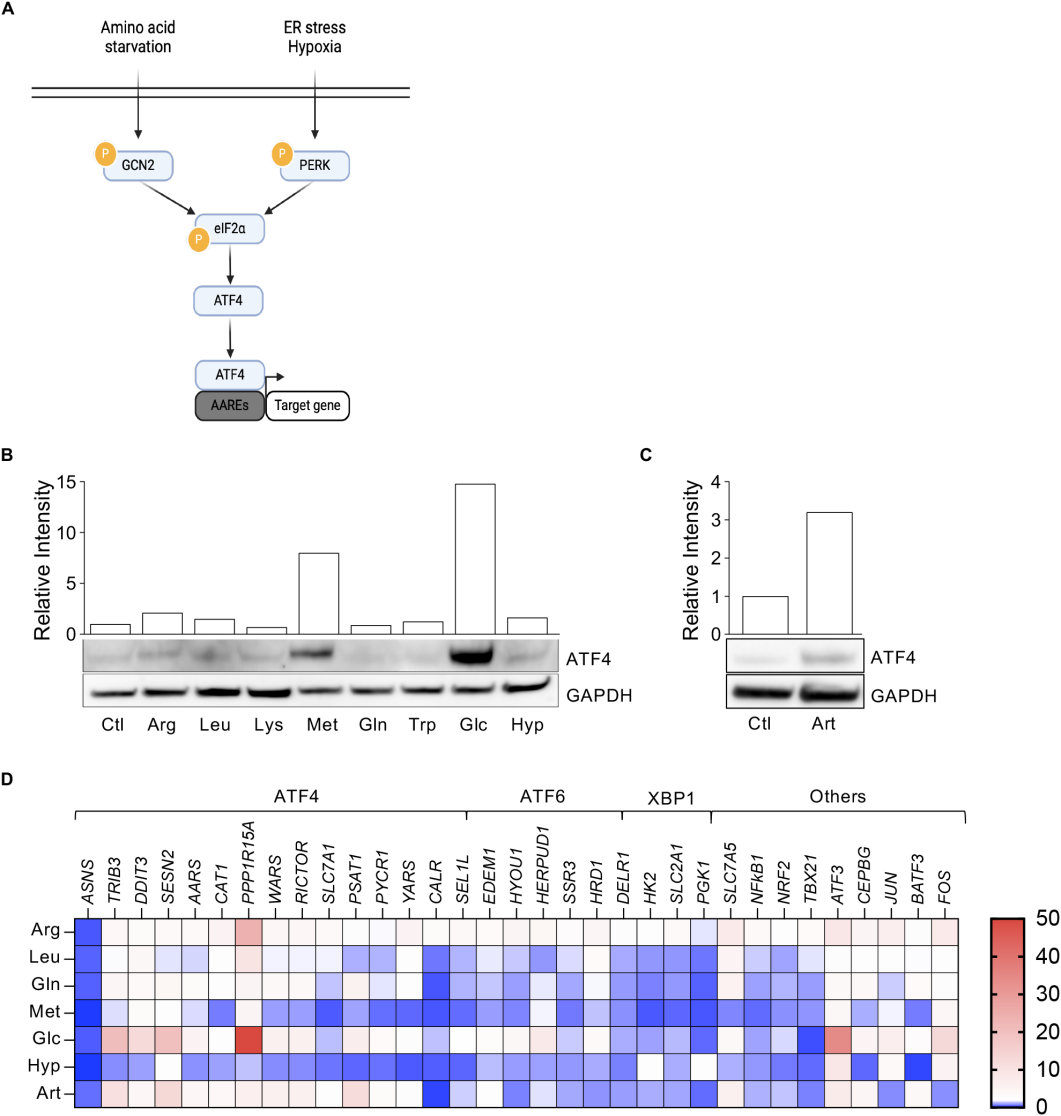
ATF4 activation in NK-92 cells. **(A)** Schematic representation of the integrated stress response (ISR) pathways leading to ATF4 expression. **(B)** Western Blot showing ATF4 expression in NK-92 cells cultured for 24h under amino acid, glucose, or O_2_ (severe hypoxia 0.1% O_2_) restriction. Histogram shows ATF4 levels normalized to GAPDH. **(C)** Western Blot showing ATF4 expression in NK-92 cells after 24h treatment with Artesunate (1μM). Histogram shows relative ATF4 levels normalized to GAPDH. **(D)** Heatmap illustrating mRNA expression of ATF4-, ATF6-, and XBP1-target genes, and genes associated with NK cells activity, in NK-92 cells after 24h under nutrient and O_2_ restriction. Data are presented as fold change relative to cells in control medium.

To explore the potential of the activating transcription factor-4 (ATF4) pathway (**Figure 1A**) in fine-tuning the activity of FDA-approved NK-92 cells for solid tumors, we first assessed ATF4 induction in these cells. To this end, we simulated nutrient-deprived tumor microenvironment (TME), including limitation of amino acids (arginine (Arg), leucine (Leu), lysine (Lys), methionine (Met), glutamine (Gln), tryptophan (Trp)) as well as glucose (Glc) restriction, alongside severe hypoxia (0.1% O_2_). Among these conditions, only Glc and Met deprivation upregulated ATF4 in NK-92 cells (**Figure 1B**), whereas most other amino acid limitations and hypoxia had minimal effects. The ER stress-inducing drug artesunate (Art) also efficiently induced ATF4 expression in NK-92 cells (**Figure 1C**). These results suggest that ATF4 activation in NK-92 cells may occur via both GCN2 and PERK signaling pathways. Consistently, Glc deprivation and Art treatment upregulated ATF4 target genes, including *Trib3, Ddit3, Sesn2, Ppp1r15a* and *Psat1*. While most amino acid shortages had only modest effects, Arg, Leu and Gln deprivation increased expression of ATF4-target genes to a limited extent. Notably, Met deprivation induced ATF4 itself without activating its downstream target genes, whereas hypoxia had no measurable effect. Furthermore, nutrient limitation and hypoxia increased the expression of ATF4 cofactors (*Atf3*, *Cebpg*) and AP-1 complex components (*Jun*, *Fos*), while downregulating several activation-related genes, including *Nfkb1, Nrf2* and *Tbx21* (**Figure 1D**).

Overall, these findings underscore glucose deprivation as a key trigger of ATF4 pathway activation in NK-92 cells and support exploiting this pathway to enhance their therapeutic potential for nutrient-limited solid tumors.

### 2xAARE-YB system drives ATF4-dependant CAR expression in NK-92 cells within the tumor microenvironment

**Figure 2:**
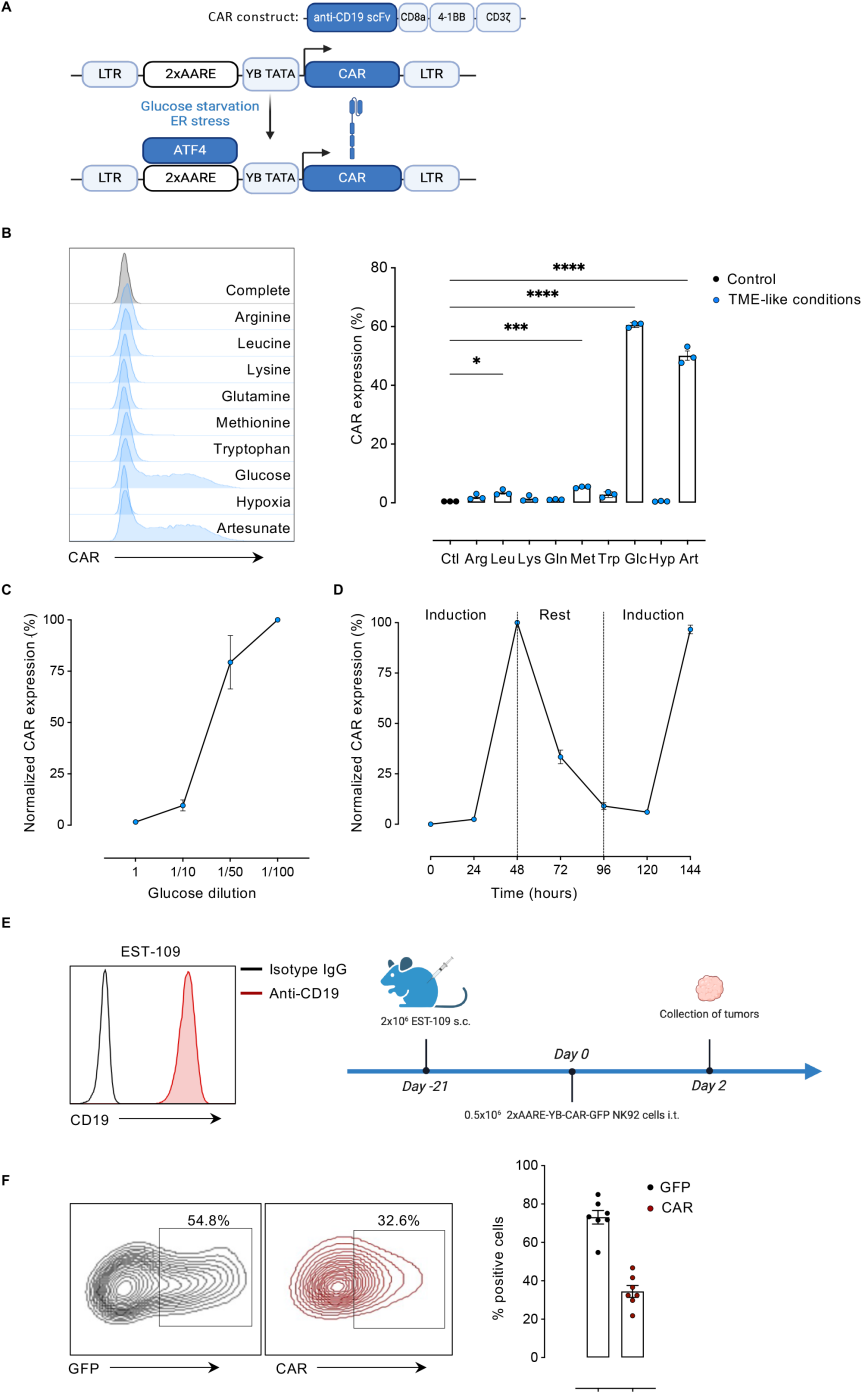
2xAARE-YB system drives ATF4-dependent CAR expression in NK-92 cells. **(A)** Schematic representation of the 2xAARE-YB-regulated CAR expression system. **(B)** Representative histogram (left) and bar plot (right) showing CAR expression in 2xAARE-YB-CAR NK-92 cells after 48h of culture under nutrient restriction, severe hypoxia (0.1% O_2_), or exposure to Artesunate (1μM). **(C)** Relative CAR expression in 2xAARE-YB-CAR NK-92 cells after 48h of culture at increasing glucose dilutions. **(D)** Time-course of relative CAR expression in 2xAARE-YB-CAR NK-92 cells under glucose-restricted conditions (Induction), followed by re-exposure to control medium (Rest), and subsequent re-restriction (Induction). In panels C and D CAR expression (%) at the highest 1/100 dilution is normalized to 100%. All results are presented as mean values ± SEM from three independent experiments. Statistical analyses were performed with one-way Anova. Asterisks represent the significance between groups and control (*p<0.05; ***p<0.001 and ****p<0.0001). **(E)** CD19 expression in EST-109 cell line against IgG isotype control (left). Schematic representation of the *in vivo* protocol: groups of NXG mice (n=7 per group) were s.c. injected with 5x10^5^ CD19^+^-EST-109. After 21 days of tumor development, mice received i.t. injection of either 2xAARE-YB-CAR or EF1α-CAR NK-92 cells. Tumors were collected two days post-injection (right). **(F)** Representative flow cytometry plots (left) and bar graphs (right) showing the percentage of (%) CAR- and GFP-positive NK-92 cells (2xAARE-YB-CAR or 2xAARE-YB-GFP) recovered from CD19^+^-EST-109 tumors. All results are presented as mean values ± SEM obtained with 7 mice per group.

Previous studies demonstrated that an ATF4-dependent inducible system, 2xAARE, can drive transgene expression (11). Building on this, we recently developed a modified version, 2xAARE-YB, which effectively restricts CAR expression in T cells under amino acid-limited TME conditions (8). Given that glucose scarcity upregulates ATF4 in NK-92 cells, we assessed whether 2xAARE-YB could similarly confine CAR expression in these cells in a glucose-dependent manner. NK-92 cells were engineered to express second generation anti-CD19-41BBz CAR under 2xAARE-YB control (**Figure 2A**). Among various TME-mimicking stressors, only glucose deprivation and artesunate treatment induced CAR expression, with no detectable leakiness under normal conditions (**Figure 2B**). CAR levels increased as glucose availability decreased and were reversible upon glucose restoration, acting as a glucose-sensitive on/off switch (**Figures 2C, D**).

To evaluate *in vivo* relevance, we implanted CD19^+^EST-109 melanoma tumors in immunodeficient NXG mice and injected intratumorally 2xAARE-YB-CAR or 2xAARE-YB-GFP NK-92 cells (i.t.) (**Figure 2E**). NK-92 cells recovered from tumors exhibited strong CAR and GFP expression (**Figure 2F**), confirming TME-dependent activation of the inducible system.

Collectively, these findings establish 2xAARE-YB as an effective ATF4-based platform for tumor microenvironment–restricted CAR expression in NK-92 cells.

### 2xAARE-YB CAR-NK-92 cells mediate cytotoxicity

**Figure 3:**
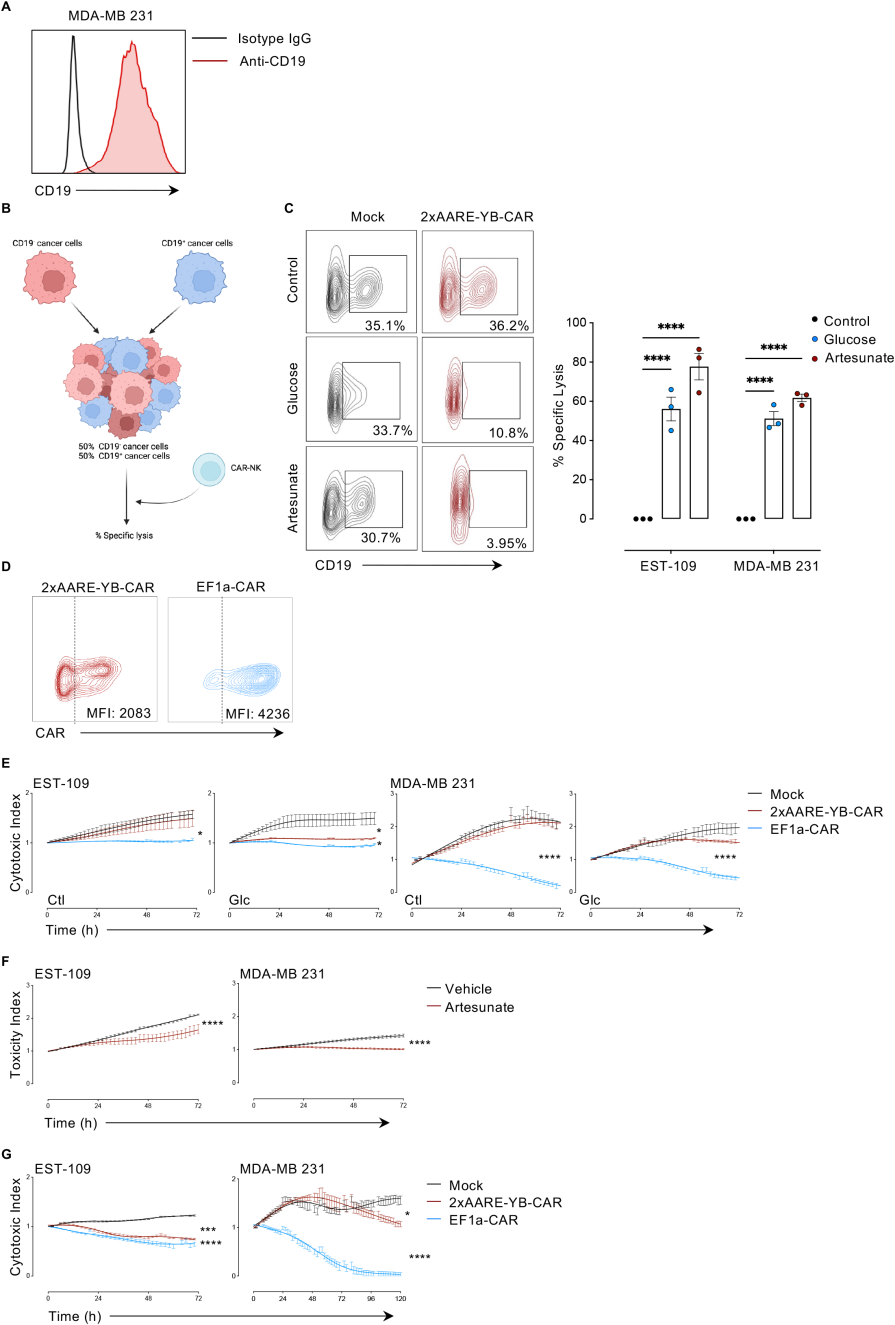
Cytotoxic potential of 2xAARE-YB CAR NK-92 cells. **(A)** CD19 expression in MDA-MB 231 cell line against IgG Isotype control. **(B)** Schematic representation of the 2D cytotoxicity assay. **(C)** Representative contour plots (left) and bar graphs (right) showing the percentage of (%) specific lysis of CD19^+^ EST-109 and CD19^+^ MDA-MB 231 tumor cells after 30 h of co-culturing with mock or 2xAARE-YB-CAR NK-92 cells at an effector-to-target (E:T) ratio of 2:1, under glucose-restricted conditions or in the presence of Artesunate (1μM). Results are presented as mean values ± SEM from three independent experiments. Statistical analyses were performed with two-way Anova. Asterisks represent the significance between groups and control (****p<0.0001). **(D)** Representative contour plot of CAR expression (MFI; geometric mean) in CAR-positive cells from 2xAARE-YB-CAR and EF1α-CAR NK-92 cells. **(E)** Time-course analysis of mock, 2xAARE-YB-CAR and EF1α-CAR NK-92 cells capacity to disrupt CD19^+^-EST-109 and CD19^+^-MDA-MB 231 spheroids under control and glucose-restricted conditions. **(F)** Size of CD19^+^-EST-109 and CD19^+^-MDA-MB 231 spheroids cultured in control medium supplemented with DMSO (vehicle) or 1μM Artesunate. **(G)** The effects of mock, 2xAARE-YB-CAR and EF1α-CAR NK-92 cells on CD19^+^-EST-109 and CD19^+^-MDA-MB 231 spheroids size overtime following exposure to Artesunate (1μM). All results are presented as mean values ± SEM from four independent experiments. Statistical analyses, two-way Anova. Asterisks represent the significant differences between groups and control (*p<0.05, ***p<0.001, ****p<0.0001).

Having established that the 2xAARE-YB system drives CAR expression in NK-92 cells under TME-like glycolytic stress, we next investigated whether this translates into effective cytotoxicity. Since NK cell activity can be influenced by HLA class I (HLA-I) on target cells (12). we used two CD19-expressing tumor models: HLA-I-negative EST-109 melanoma (13), and HLA-I-positive MDA-MB-231 breast cancer cells (14) (**Figures 2E**, **3A**, respectively), tested in both 2D and 3D-spheroid assays.

In 2D co-cultures, mock or 2xAARE-YB-CAR NK92 cells were incubated with a 1:1 mixture of CD19^-^ and CD19^-^ tumor cells under glucose deprivation or artesunate treatment (**Figure 3B**). 2xAARE-YB-CAR NK-92 cells selectively lysed CD19^-^ cells, irrespective of HLA-I status (**Figure 3C**). In 3D assays, CD19^+^EST-109 or CD19^+^MDA-MB 231 spheroids were co-cultured with mock, 2xAARE-YB-CAR, or constitutive EF1α-CAR NK-92 cells under normal or glucose-deprived conditions (**Figure 3D**). We then measured the reduction in GFP-positive spheroid size, as a dynamic and non-invasive readout to monitor changes in tumor spheroid integrity over time commonly accepted as a proxy for cytotoxic activity. Under normal conditions, only EF1α-CAR NK-92 cells reduced spheroid size. In contrast, glucose limitation enabled 2xAARE-YB-CAR NK-92 cells to effectively reduce spheroid size in both tumor models, consistent with inducible cytotoxic activity of 2xAARE-YB-CAR NK-92 cells (**Figure 3E**). Artesunate alone induced a modest reduction in spheroids size, but its combination with 2xAARE-YB-CAR NK-92 cells further enhanced spheroid disruption, with a greater reduction of EST-109 spheroids at 72h and MDA-MB-231 spheroids at 144h (**Figures 3F, G**). Although artesunate possesses intrinsic cytotoxic activity and could, in principle, represent a potential confounding factor, the markedly enhanced effect observed exclusively in combination with 2xAARE-YB-CAR NK-92 cells indicates that direct artesunate toxicity toward NK-92 cells is unlikely to account for the observed effects. Notably, under glucose deprivation and artesunate treatment, 2xAARE-YB-CAR NK-92 cells were as effective as EF1α-CAR NK-92 cells in reducing the size of HLA-I^-^CD19^-^ EST-109 spheroids, but exhibited slightly reduced efficacy against HLA-I^-^CD19^-^ MDA-MB-231 spheroids (**Figures 3E, G**). This differential response may reflect differences in HLA-I expression, surface antigen density, or tumor heterogeneity.

Together, these results suggest that the 2xAARE-YB system can confer effective, stress-inducible CAR-NK-92 cell cytotoxicity against a range of solid tumors, while minimizing activity under physiological conditions, highlighting its potential for safer and more targeted immunotherapy.

## Discussion

To address the challenges of CAR therapy in solid tumors, we recently validated the pathophysiologically regulated 2xAARE-YB system as an innovative strategy to enhance CAR-T cell safety and fitness (8). Here, we extend its application to the FDA-approved NK-92 cells, demonstrating that 2xAARE-YB enables selective, functional CAR expression under tumor-specific conditions or upon exposure to the clinically approved drug artesunate. This versatile system, incorporating a built-in safety mechanism, offers a promising strategy for advancing the development of CAR-NK-92 cells therapies for solid tumors.

Few studies have engineered TME-sensitive systems to restrict CAR expression in NK cells to tumors. One strategy employed IFNγ-, TNF- and hypoxia-sensitive promoters, which can be effective in inflamed “hot” tumors but often exhibit leaky expression under physiological conditions and limited efficacy in poorly inflamed “cold” tumors (15, 16). In contrast, our approach harnesses a universal feature of TME, glucose deprivation, to control CAR expression and minimize off-tumor activity. While the 2xAARE system has primarily been associated with amino acid limitation (8, 11), we found that in NK-92 cells, activation is mainly driven by glucose scarcity, likely reflecting their heavy reliance on glycolysis (17).

Amino acid deprivation, except for Met starvation, modestly upregulated ATF4 target genes but was insufficient to trigger satisfactory CAR expression via 2xAARE-YB. NK-92 cells are highly dependent on mTOR signaling and Met metabolism and exhibit elevated basal turnover of S-adenosylmethionine (SAM), rendering them particularly sensitive to disruptions in epigenetic regulation. Consequently, amino acid deprivation and activation of GCN2–eIF2α–ATF4 integrated stress response in NK-92 may impair biosynthetic capacity and alter epigenetic permissiveness, limiting sustained transcriptional programs despite ATF4 translation.

Collectively, these findings suggest that in NK-92 cells, amino acids deprivation, particularly Met starvation, permits ATF4 accumulation but restricts its transcriptional activity. In contrast, glucose deprivation, through activation of PERK-eIF2α stress response, generates ATF4 that is both present and transcriptionally competent, enabling activation of downstream targets and CAR expression. This glucose-specific responsiveness offers a tailored strategy for tissue-specific CAR expression in NK-92 cells infiltrating malignant tumors.

Although glucose deprivation is reported to suppress innate NK cell cytotoxicity (18), our data obtained in 2D models indicate that CAR signaling upon antigen engagement can override this inhibition in NK-92 cells, enabling cytotoxic activity. In 3D spheroid models under glucose-limited conditions, 2xAARE-YB-CAR-NK-92 cells significantly reduced the size of both CD19^+^EST-109 and CD19^+^MDA-MB 231 spheroids, with greater efficacy observed in the former, supporting the functionality of glucose-regulated CAR activity irrespective of HLA expression. While reduced GFP fluorescence is not a direct qualification of tumor cell killing, it is commonly used as an indicator of spheroid disruption and reduced tumor cell viability. Collectively these results support the cytotoxic potential of 2xAARE-YB-CAR-NK-92 cells under conditions that partially recapitulate key features of the tumor microenvironment, although their therapeutic effect may be more pronounced in HLA-I–negative tumors than in HLA-I–positive tumors.

Dual hypoxia-sensing systems have been used to restrict CAR expression in T cells, but concerns remain regarding unintended activation in physiologically hypoxic tissues (19, 20). Similarly, while the 2xAARE-YB system could theoretically respond to low-glucose environment such as the brain (21), our data revealed that significant CAR expression only occurs under severe glucose deprivation, and the blood-brain barrier would likely limit NK infiltration (22). Importantly, CAR expression was fully downregulated within two days of glucose restoration. Although transient expression may pose off-tumor risks, CD8^-^ T cells are known to halt migration upon antigen recognition (23), and CAR-NK-92 cells may exhibit similar behavior, reducing the likelihood of unintended tissue damage.

The TME is inherently dynamic, with fluctuating glucose levels that could impact 2xAARE-YB-driven CAR expression. However, the system incorporates a built-in switch via drug inducibility with artesunate, providing a fail-safe mechanism to maintain CAR-NK-92 activity under variable metabolic conditions. Artesunate may also synergies with CAR-mediated cytotoxicity. Although artesunate induces systemic CAR expression, this potential toxicity would likely be rapidly reversible upon drug withdrawal, preserving a favorable safety profile.

While our previous work established the 2xAARE-YB dual-modal regulatory system in CAR-T cells (8), its application to NK-92 cells represents a distinct therapeutic advancement. Unlike T cells, which predominantly respond to amino acid deprivation, NK-92 cells display a pronounced metabolic sensitivity to glucose limitation, enabling CAR induction under conditions that closely mirror a key metabolic constraint of the tumor microenvironment. This strategy supports the development of “off-the-shelf” CAR-NK products, providing a scalable, standardized allogeneic platform with inherently lower risk of graft-versus-host disease compared with T-cell–based approaches, while maintaining context-dependent control of CAR expression.

## Conclusion

Our study demonstrates that the 2xAARE-YB system provides a versatile dual-induction platform for further development of CAR-NK-92 cell therapies targeting solid tumors with the potential to reduce off-target risks. By exploiting nutrient deprivation and offering drug-inducible control through artesunate, this platform combines antigen-specific cytotoxicity with environmental regulation, providing a flexible and potentially safer approach to solid-tumor immunotherapy. These findings highlight the translational potential of 2xAARE-YB CAR-NK-92 cells and support their continued development as adaptable, off-the-shelf cellular therapies for solid tumors applications.

## Data Availability

The original contributions presented in the study are included in the article/**Supplementary Material**, further inquiries can be directed to the corresponding author/s.
