# Supplementary material for: Tunable CAR-NK-92 activity in the tumor microenvironment via a dual ATF4-responsive circuit
Source: Front Immunol. 2026 Mar 5;17:1792164. doi: 10.3389/fimmu.2026.1792164 (PMC12999950; doi:10.3389/fimmu.2026.1792164)
Supplement: Supplementary file 1 [file DataSheet1.pdf]

Tunable CAR-NK-92 activity in the tumor microenvironment via a dual ATF4-responsive circuit

Enzo Manchon[1†], Nell Hirt[1††], Aravindhan Soundiramourty[2], Benjamin Versier[2], Yves Christen[2], Dominique Charron[1,2], Jacques Mallet[2], Nabila Jabrane-Ferrat[3], Che Serguera[2*], Reem Al-Daccak[1*‡]

Supplementary Information

Detailed Methods

Tables 1-3

Methods

Cell lines

NK-92 (ACC-488) from DSMZ were engineered to constitutively express IL-15 and maintained *in vitro* in Roswell Park Memorial Institute medium (RPMI-1640, Gibco) supplemented with 20% Fetal Bovine Serum (FBS; Gibco), 1X GlutaMAX (Gibco) and 1X penicillin-streptomycin (Gibco) (NK-92 medium). MDA-MB 231 (HTB-26) breast cancer cells, from ATCC, and ESTDAB-109 (UKRV-Mel-2, referred to as EST-109) melanoma cells, a gift from Federico Garrido Torres-Puchol (Granada University), were maintained in RPMI-1640 supplemented with 10% FBS, 1X GlutaMAX and 1X penicillin-streptomycin (cancer cell medium). Cell lines were frequently tested for Mycoplasma using MycoBlue Mycoplasma Detector (Vazyme).

CAR construct and lentiviral production

Self-inactivated (SIN) lentiviral vector plasmid (pLV), containing inducible cassettes of interest, were cloned by NEBuilder HiFi DNA Assembly (New England Biolabs) insertion of the synthetized or PCR amplified (2xAARE-YB-transgene (± polyA)) cassettes into destination vector containing restriction sites within the lentiviral genome. The cDNA sequences of CAR anti-CD19 were cloned under the control of an EF1a or 2xAARE-YB promoter. Recombinant particles were obtained as described (Zennou, V. *et al. Nat. Biotechnol.* 2001). Non-replicative lentiviral particles (LV) were produced in HEK-293T cells (ATTC-CRL-11268), as per standard second generation protocol (Zennou, V. *et al. Nat. Biotechnol.* 2001). Briefly, cells were co-transfected with the pLV and trans-complementation plasmids p8.91 (packaging plasmid encoding Gag-Pol, Tat, and Rev) and pVSVg (VSV-G envelope expression plasmid). Briefly, 1.2×10^5 cells/cm^2 were seeded 24 h prior co-transfection. For each 10^6 seeded cells, 1.5 µg of pLV or p8.9, and 0.75 µg of pVSVg plasmid were co-transfected using calcium phosphate method or jetOPTIMUS (Polyplus-Sartorius) according to manufacturer’s protocol. Media were changed 5 h later and LV-containing media were collected 48 h post transfection. For *ex vivo* T cell engineering, supernatant was passed through a 0.2 µm filter, and ultracentrifuged at 60 000 g and 4°C for 1.5 h. Pelleted particles were concentrated 1000 times in PBS and stored at -80°C. Recombinant particles were titrated by measurement of p24 viral protein with ELISA (Kit ref. 0801008, Zeptometrix). For all experiments, 1pg of p24 is equivalent to 100 transduction units (TU).

Lentiviral transduction

To obtain NK-92 cells constitutively producing IL-15, cells were transduced with a “TRAP-IL-15” lentiviral vector, and to obtain NK-92 expressing second-generation anti-CD19-41BBz CAR under the control of 2xAARE-YB system or EF1a promoter, cells were transduced with “2xAARE-YB-CAR” or “EF1a-CAR” lentiviral vectors. NK-92 cells were incubated for 16 h in

NK MACS medium (Miltenyi Biotec) supplemented with 1X NK MACS supplement, 10% Fetal Bovine Serum (FBS; Gibco), 1000U/ml recombinant human IL-2 (Miltenyi Biotec), 1X GlutaMAX (Gibco) and 1X penicillin-streptomycin (Gibco) (transduction medium) at a concentration of 200.000 cells/ml. Then, were seeded in retronectin-coated plates (20μg/ml) at a density of 200.000 cells/ml in transduction medium supplemented with 2μM (5Z)-7-Oxozeaenol (Sigma-Aldrich). Lentivirus concentrates were added in the medium with a multiplicity of infection (MOI) of 20 and plates were spinoculated for 70 min at 32°C before incubation at 37°C. After 16 h, cells were washed with fresh NK-92 medium and expanded.
To obtain EST-109 and MDA-MB 231 cells expressing the CD19 antigen, cells were transduced with a “TRAP-CD19” lentiviral vector (multiplicity of infection (MOI) of 10) carrying the fusion gene containing splice acceptor sequences, T2A (autocatalytic peptide), the CD19 cDNA, P2A (autocatalytic peptide), and blasticidin-S deaminase. This vector allows selecting blasticidin-resistant cells expressing the CD19 antigen under a cellular promoter, ensuring the stability of the transgene expression overtime. Transduced blasticidin-resistant cells were then labeled with an anti-CD19 antibody, sorted for high expression of CD19 antigen and amplified. The stability of CD19 expression by EST-109 and MDA-MB 231 cells was regularly checked.

In vitro studies in solid tumor microenvironment-like conditions

Transduced cells or untransduced cells were washed twice in PBS and were resuspended in different single nutrient-restricted media (RPMI-1640 depleted in arginine, leucine, lysine, methionine, glutamine, tryptophan, or glucose) supplemented with 5% FBS (Gibco), 1X GlutaMAX (Gibco; except for glutamine-restricted condition), 1X penicillin-streptomycin (Gibco), and 1/1000 or 1/100 RPMI concentration of the restricted amino acid or glucose, respectively (Nutrients concentrations are listed in Supplementary Table S1) or a control medium (RPMI supplemented with 10% FBS, 1X GlutaMAX and 1X penicillin-streptomycin at a concentration of 200.000 cells per ml for 24 or 48 h. For hypoxic conditions, cells were incubated in control medium in a hypoxia incubator chamber (Stemcell technologies) purged at 25L/min for 5 min with gas containing 0.1% O_2, 5% CO_2 and nitrogen as a balance. Under all experimental conditions, cells were seeded at a concentration of 200.000 cells/ml and incubated for 24 or 48 h.

Flow cytometry

NK-92 cells were stained with fluorophore-conjugated cell-surface antibodies (1/100) in FACS buffer (PBS + 0.5%FBS) for 15 min at room temperature, followed by two washes. For CAR detection, cells were first incubated with CD19 CAR Detection Reagent (1/500) (Miltenyi Biotec) for 15 min, stained at room temperature, then washed twice with FACS buffer before proceeding to the abovementioned cell-surface staining in addition to the anti-biotin antibody,

REAfinity (clone REA746) (1/200) (Miltenyi Biotec) alongside other surface markers. Samples were acquired on a BD LSR Fortessa running FACS Diva software, and analyzed using FlowJo v.10.9 software. Antibodies used are listed in Supplementary Table S2.

Quantitative reverse transcriptase PCR

Total RNA was extracted from NK-92 cells exposed to various conditions using the RNeasy Plus Mini Kit (Qiagen, France). cDNA was synthesized from 2 μg of RNA using the High-Capacity cDNA Reverse Transcription Kit (Thermo Fisher Scientific, France). qPCR was performed with 5 ng cDNA using Fast SYBR Green Master Mix (Thermo Fisher Scientific) and specified primers (Supplementary Table S3) on a CFX384 Touch Real-Time PCR Detection System (Bio-Rad Laboratories, France). Each independent experiment included three technical replicates. Gene expression was normalized against *Ubc* (Ubiquitin C) and analyzed using the $2^{-\Delta\Delta Ct}$ method, with results expressed as fold change relative to control.

Immunoblotting

NK-92 cells were lysed in RIPA buffer (Thermo Fisher scientific), and proteins separated on 10% SDS-PAGE under reducing conditions, then transferred to nitrocellulose membranes using ™iBlot™2 (Invitrogen, France). Membranes were blocked in TBST with 2% BSA, hybridized overnight with primary antibodies against ATF4 (1/1000) (clone D4B8, Cell Signaling technology) or GAPDH (1/1000) (clone 6C5, Abcam), followed by HRP-conjugated secondary antibody (1/3000) (Bio-Rad). Signal was detected with SuperSignal West Pico PLUS Chemiluminescent Substrate reagents (Thermo Fisher scientific) and imaged with ImageQuant™ LAS 4000/4010 (GE Healthcare).

2D cytotoxicity assay

5.000 $CD19^-$ and 5.000 $CD19^+$ EST-109 melanoma or MDA-MB 231 breast cancer cells were co-cultured with 20.000 CAR-NK-92 cells in 200 μl control media, glucose-restricted media or control media supplemented with Artesunate (1μM) in flat bottomed 96-well plates for 48 h. Target cells were then detached using 0.05% Trypsin-EDTA (Gibco) before being acquired on BD Canto II (BD biosciences) running FACS Diva software and analyzed using FlowJo (10.8.1) software. Specific lysis (%) was calculated as (1-(control ratio/experimental ratio) x 100) where the control ratio corresponds to target cells co-cultured with untransduced (mock) NK-92 cells.

Spheroid formation

5.000 EST-109 melanoma cells or MDA-MB 231 breast cancer cells were seeded in 15 μl of spheroid media (RPMI supplemented with 5% FBS, 1X GlutaMAX, 1X penicillin-streptomycin

and 2.5% Matrigel Basement Membrane Matrix (Corning)) in ultra-low attachment (ULA) round-bottomed 96-well plates (PHCBI) and were then spinoculated at 200 g for 3 min. Spheroid were grown in spheroid media for 4 days before being used for further experiments.

3D Incucyte cytotoxicity assay

In this experimental context, cancer cells were constitutively expressing GFP. Following the formation of EST-109 melanoma or MDA-MB 231 breast cancer spheroids, these spheroids were rinsed once in PBS and then transferred to round-bottomed 96-well plates containing 200 μl of control media, glucose-restricted media or control media supplemented with Artesunate (1 μM). Subsequently, 20.000 untransduced (mock), 2xAARE-YB-CAR, or EF1a-CAR T cells were added to each spheroid. Fluorescence was assessed every 2 h using a 10x objective on the Incucyte system (Essen Bioscience). The size of the largest GFP object was determined using Incucyte software (Essen Bioscience). All data were normalized to the initial time point and represented as the fold change of the largest GFP object over time.

***Mice and in vivo* experiments**

Experiments using animals were approved by the Institut de Recherche Saint-Louis Animal Care and Use Committee (committee Paris-Nord/n°121 (agreement APAFIS #43896-2023062108019955 v6). Male NXG (NOD-*Prkdc*^{scid} -*IL2rg*^{Tm1}) mice were purchased from Janvier labs and maintained in pathogen-free conditions.

$CD19^+$-EST-109 cells ($2x10^6$ cells in PBS) were subcutaneously (s.c.) injected into 6-8 weeks old male mice. The indicated dose of mock, 2xAARE-YB-CAR or 2xAARE-YB-GFP NK-92 cells was intratumorally (i.t.) injected in 50 μl of PBS. At endpoints, mice were euthanized and tumors were collected and kept in RPMI supplemented with 10% FBS. Tumors were then minced and single cell suspensions were obtained after 1 h digestion with 250U/ml Collagenase IV (Gibco) and 0.1mg/ml DNase I (Gibco) at 37°C. After digestion cells were filtered through a 70 μm cell strainer prior to subsequent antibody staining and flow cytometry analysis.

Statistics

Statistical analyses were conducted with GraphPad Prism 10. Details regarding sample size and plot descriptions can be found in the corresponding Figure legends. To assess statistical variances between two groups, either unpaired Student's t-test or Mann-Whitney U test was employed. Statistical differences of three or more groups were analyzed using one-way or two-way ANOVA with the appropriate multiple comparison tests. Significance is denoted by p-values less than 0.05.

Supplementary Table S1. Amino acid concentration in control medium and single amino acid-restricted media

Component	RPMI concentration (μM)	*Nutrient-restricted media* (μM)
L-Arginine	1150	1.150
L-Leucine	380	0.38
L-Lysine	220	0.22
L-Methionine	100	0.1
L-Glutamine	2055c	2.055
L-Tryptophan	25	0.025
D-Glucose	11111	111.11

Supplementary Table S2. Antibodies and reagents used for flow cytometry staining

Reagent	Manufacturer	Catalog number
CD56 Antibody, anti-human, APC, REAfinity (clone REA196)	Miltenyi Biotec	Catalog No: 130-113-310
CD45 Antibody, anti-human, PE, REAfinity (clone REA747)	Miltenyi Biotec	Catalog No: 130-110-632
BD Horizon BV650 Mouse Anti-Human CD19 (clone SJ25C1)	BD biosciences	Catalog No: 563226
Biotin antibody, PE-Vio 770, REAfinity (clone REA746)	Miltenyi Biotec	Catalog No: 130-110-953
R-Phycoerythrin Streptavidin	Jackson Lab	Catalog No: 016-110-084
BD Pharmingen 7-AAD	BD biosciences	Catalog No: 559925
LIVE/DEAD Fixable Lime (506) Viability Kit	Thermo Fisher	Catalog No: L34990

Supplementary Table S3. Sequences of primers used for quantifying mRNA expression in NK-92 cells using RT-qPCR.

Gene	Sequences
ASNS	5’ atcactgtcgggatgtaccc 3’; 5’ cttcaacagagtggcagcaa 3’
CAT1	5’ gccattgtcatctccttcctg 3’; 5’ caaacagagacggcctgatg 3’
TRIB3	5’ aactggcatccttgagctgacaac 3’; 5’ aagggattgtccttggacagaggt 3’
DDIT3	5’ tgccaatgatgtgaccctcaatcc 3’; 5’ ttttgtctactccaagccttcccc 3’
PPP1R15A	5’ tgagactcccctaaaggccagaaa 3’; 5’ agacagccaggaaatggacagtga 3’
WARS	5’ gaaaggcattttcggcttca 3’; 5’ cagcctggatggcagga 3’
RICTOR	5’ agtgaatctgtgccatcgagt 3’; 5’ agtagagctgctgccaaacc 3’
CHAC1	5’ tggattttcgggtacggctc 3’; 5’ acttcagggccttgcttacc 3’
SLC7A1	5’ accttctgcattgtgaccgt 3’; 5’ cagcatccacacagcaaacc 3’
SESN2	5’ acttccgccactcagagaag 3’; 5’ gtcaggtcatgtagcgggtg 3’
PSAT1	5’ gaattgctagctgttccagaca 3’; 5’ tcagcacaccttcctgcttt 3’
PYCR1	5’ tgccttgcatgtgctggagagt 3’; 5’ gcttcaccttgtccaggatggt 3’
PHGDH	5’ acgtgtttacggaagagccg 3’; 5’ cccttcaccatgtccacgaa 3’
AARS	5’ ggaccatcactgtggcact 3’; 5’ cggagaatccgtctcaacac 3’
YARS	5’ aaggactttgctgctgaggt 3’; 5’ tccagcaacttgttcagtgc 3’
CALR	5’ agcagaacatcgactgtggg 3’; 5’ ccacagatgtcgggaccaaa3’
SEL1L	5’ cctgaggcacgatctggatttta 3’; 5’ aataccaagtgggtggagagcttc 3’
EDEM1	5’ gtggctgagcaggaactttaga 3’; 5’ aataggagctggatgctgggaatg 3’
HYOU1	5’ gaagatgcagagcccatttc 3’; 5’ tctgctccaggacctcctaa 3’
HERPUD1	5’ tgagcacagcatggcttgtctt 3’; 5’ agcctccaacagctacagcacaaa 3’
SSR3	5’ gttcatcgtgtctgccatcc 3’; 5’ catcctccctcttctgtgctac 3’
HRD1	5’ cctgtggcatttctgcgtcttact 3’; 5’ caggcattcctttccctgagttgt 3’
HK2	5’ tcccctgccaccagacta 3’; 5’ tggacttgaatcccttggtc 3’
SLC2A1	5’ ggttgtgccatactcatgacc 3’; 5’ cagataggacatccagggtagc 3’
PGK1	5’ ctgtggcttctggcatacct 3’; 5’ cgagtgacagcctcagcata 3’
SLC3A2	5’ gcttccaagaatgctgaggtt 3’; 5’ gcagtcggatcaattgaggt 3’
SLC7A5	5’ ccgtctacttcttcggggtc 3’; 5’ cttctgacacaggacggtcg 3’
NFATC1	5’ tggaccagttgtacctggatg 3’; 5’ gtgctccaatgtggcaacta 3’
NFKB1	5’ cctggaaccacgcctcta 3’; 5’ ggctcatatggtttcccattta 3’
NRF2	5’ gagacaggtgaatttctcccaat 3’; 5’ tttgggaatgtgggcaac 3’
TBX21	5’ gcctaccagaatgccgagatta 3’; 5’ ggactcaaagttctcccggaat 3’
ATF3	5’ cgctggaatcagtcactgtcag 3’; 5’ cttgtttcggcactttgcagctg 3’

FOS1L	5’ ggaggaaggaactgaccgactt 3’; 5’ ctctaggcgctccttctgcttc 3’
CEBPG	5’ gcttacagcaggttcctcagct 3’; 5’ cgttgccgatactcgtcactgt 3’
JUN	5’ ccttgaaagctcagaactcggag 3’; 5’ tgctgcgttagcatgagttggc 3’
BATF3	5’ accgagttgctgctcagagaag 3’; 5’ aggtgcttcagctcctctgtca 3’
FOS	5’ gcctctcttactaccactcacc 3’; 5’ agatggcagtgaccgtgggaat 3’
GUSB	5’ tgcgtagggacaagaaccac 3’; 5’ gggaggggtccaaggatttg 3’
